# Optimizing Management of Testicular Torsion at Bronglais General Hospital: Assessing the Testicular Workup for Ischemia and Suspected Torsion (TWIST) Score and Ultrasound for Effective Time Intervention

**DOI:** 10.7759/cureus.97469

**Published:** 2025-11-21

**Authors:** Sami E Mohammed, Samy R Mohamed, Michael Aniah, Mohamed A Ali, Arinze Chukwuonwe, Subhiksha Kanagaraj

**Affiliations:** 1 Urology, Glangwili General Hospital, Carmarthen, GBR; 2 General Surgery, South Tyneside District Hospital, South Shields, GBR; 3 General Surgery, Bronglais General Hospital, Aberystwyth, GBR; 4 Surgical Oncology, National Cancer Institute, Cairo, EGY

**Keywords:** assessment tools for testicular torsion, rural hospital setting, testicular torsion, testicular torsion management, testicular workup for ischemia and suspected torsion (twist), twist score assessment, ultrasound availability

## Abstract

Background: Testicular torsion (TT) is a urological emergency requiring timely surgical intervention to prevent testicular loss. Different assessment approaches were suggested to improve overall patient outcomes, as the Testicular Workup for Ischemia and Suspected Torsion (TWIST) score, which has been integrated with ultrasound findings to enhance diagnostic accuracy in suspected cases of testicular torsion. Additionally, Doppler ultrasound is recommended in accordance with recently updated national and international guidelines.

Methodology: This is a retrospective study conducted at Bronglais General Hospital (BGH), Aberystwyth, United Kingdom, from January 2019 to January 2024 of patients who had undergone scrotal exploration for suspected TT. A total of 34 patients were included in the study.

Objective: The study aims to review the management of all patients who present to BGH with suspected “testicular torsion” as per available national & international guidelines, aiming to develop a local protocol. Also, the study aims to investigate the potential causes of the delay in management of patients presented with scrotal pain and to structure a local management pathway for patients presented with scrotal pain at BGH.

Results: Out of 34 patients in our study, 67.64% (n=23) were in the age group of 11 to 20 years. Only 38% (n=13) had confirmed TT. Of these 13 patients, 69.23% (n=9) of the testes were viable, and in 30.77% (n=4), the testis was not viable. The TWIST score was applied in only 6% (n=2) of cases. Ultrasound was performed in only 9% (n=3) of cases.

Conclusion: To improve surgical outcomes for scrotal pain exploration and reduce delays in intervention, it is important to address existing documentation gaps and the inconsistent use of diagnostic tools such as the TWIST score and ultrasound, even though TT is generally managed in a timely manner at BGH. However, a local protocol has been developed that includes direct referral to the on-call surgical registrar to further decrease time to theatre. It also includes the TWIST score to guide diagnosis. Raising awareness of children and their carers/parents is of paramount importance. A proposed pathway for the management of patients with scrotal pain and suspected of TT at BGH was developed and will be followed up on in future practice.

## Introduction

Testicular torsion (TT) is a urological emergency requiring timely surgical intervention to prevent testicular loss. Management relied on clinical judgement, with the earliest surgical exploration being the approach to minimise ischaemic loss [1]. However, relatively new tools for assessing risk, such as the Testicular Workup for Ischemia and Suspected Torsion (TWIST) score and Doppler scrotal ultrasound (DUS), have improved the accuracy of diagnosis of TT, leading to accelerated scrotal explorations and ensuring that cases get treated on time [2,3]. 

Challenges persist in balancing rapid expert surgical referral with diagnostic precision on suspected TT. This study looks at how things are done now by reviewing past cases and suggests a better process that combines the assessment by more expert doctors and careful use of ultrasound to achieve better results [4].

This article was previously presented as a meeting abstract at the 2025 Association of Surgeons of Great Britain and Ireland (ASGBI) International Surgical Congress EICC, Edinburgh, Scotland, on May 13, 2025 [5].

## Materials and methods

Study design and setting

This retrospective cohort study was conducted at Bronglais General Hospital (BGH), a rural district general hospital in Aberystwyth, United Kingdom. The study reviewed all male patients who underwent scrotal exploration for suspected TT between January 2019 and January 2024.

Study population and sample size

A total of 34 patients were identified from the hospital's information department using procedural codes for scrotal exploration. All patients who underwent surgery for suspected TT within the study period were included. There were no exclusion criteria.

Data collection and study measures

Data were collected retrospectively from patients' records, including operation notes, clinic letters, and nursing charts. The primary data points collected were patient demographics, clinical presentation (including symptoms and signs), and documentation of the TWIST score. The TWIST score is a clinical tool used to assess the likelihood of testicular torsion in cases of acute scrotal pain. The TWIST score (range 0-7) comprises hard testis (2 points), testicular swelling (2 points), nausea and vomiting (1 point), high-riding testis (1 point), and absent cremasteric reflex (1 point) [2]. Patients were classified into three groups: 1. Low risk (0-2 points): Unlikely torsion; consider alternative diagnoses. 2. Intermediate risk (3-4 points): Ambiguous cases; ultrasound recommended. 3. High risk (5-7 points): There is a high probability of torsion, needing immediate surgery without prior imaging [6]. Additionally, the findings of DUS include time points from arrival to decision-to-operate and to surgery, intraoperative findings (viability of the testis), and the final diagnosis.

The primary outcome was to assess the management of suspected TT against national guidelines (Getting It Right First Time (GIRFT) [7], National Confidential Enquiry into Patient Outcome and Death (NCEPOD)) [8] to develop a local protocol. Secondary outcomes included the diagnostic accuracy of scrotal exploration (confirmed TT vs. alternative diagnoses) and an analysis of causes for delayed intervention.

Ethical considerations

This study is a retrospective clinical audit; therefore, formal ethical approval was not required as per institutional policy. Patient consent is not needed for the same reason. All data were anonymised during collection and analysis to maintain patient confidentiality.

Statistical analysis

Data analysed by using IBM SPSS Statistics for Windows, version 21 (released 2012; IBM Corp., Armonk, New York, United States). Descriptive statistics (graphs, tables, percentages) are used to summarise the data. No adjustments were applied for multiple comparisons due to the observational nature of the study.

## Results

**Figure 1:**
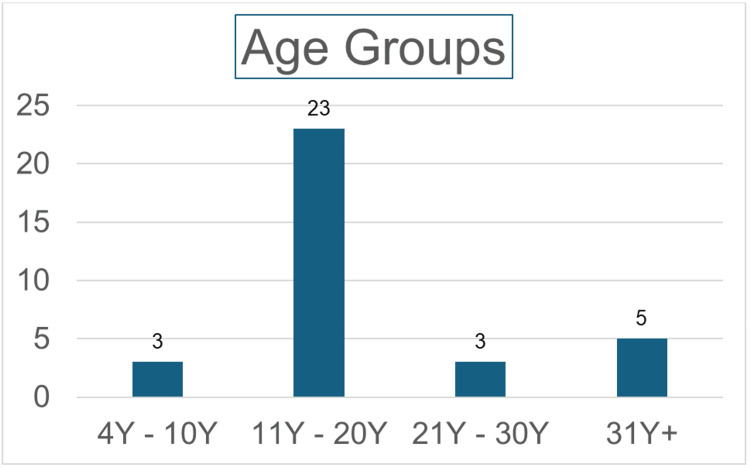
The distribution of patients as per their age group y: years

Thirty-four patients underwent scrotal exploration from January 2019 to January 2024 at BGH; 67.64% (n=23) were in the age group of 11 to 20 years (Figure 1).

Diagnostic accuracy

**Figure 2:**
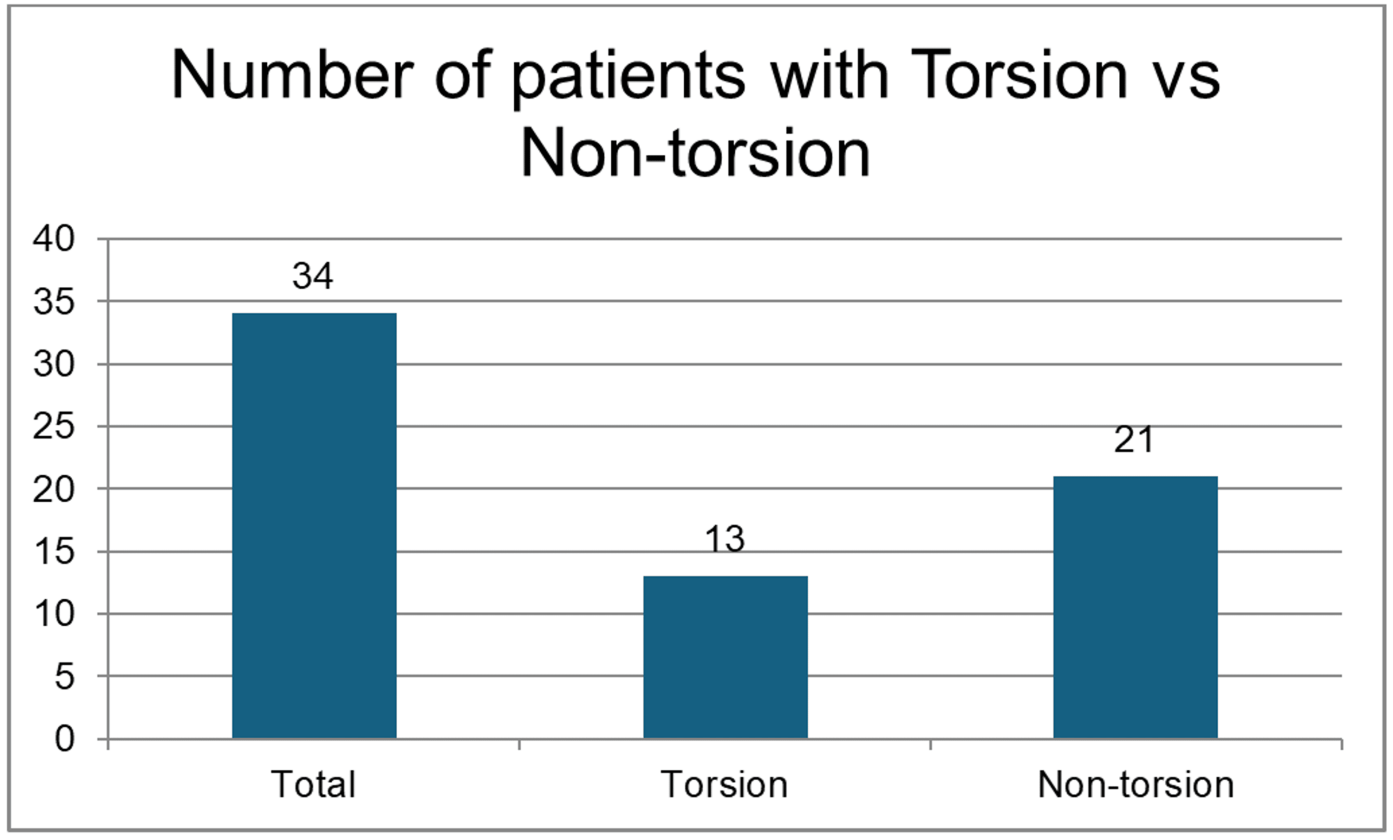
Distribution of patients as per their condition: torsion vs. non-torsion

**Figure 3:**
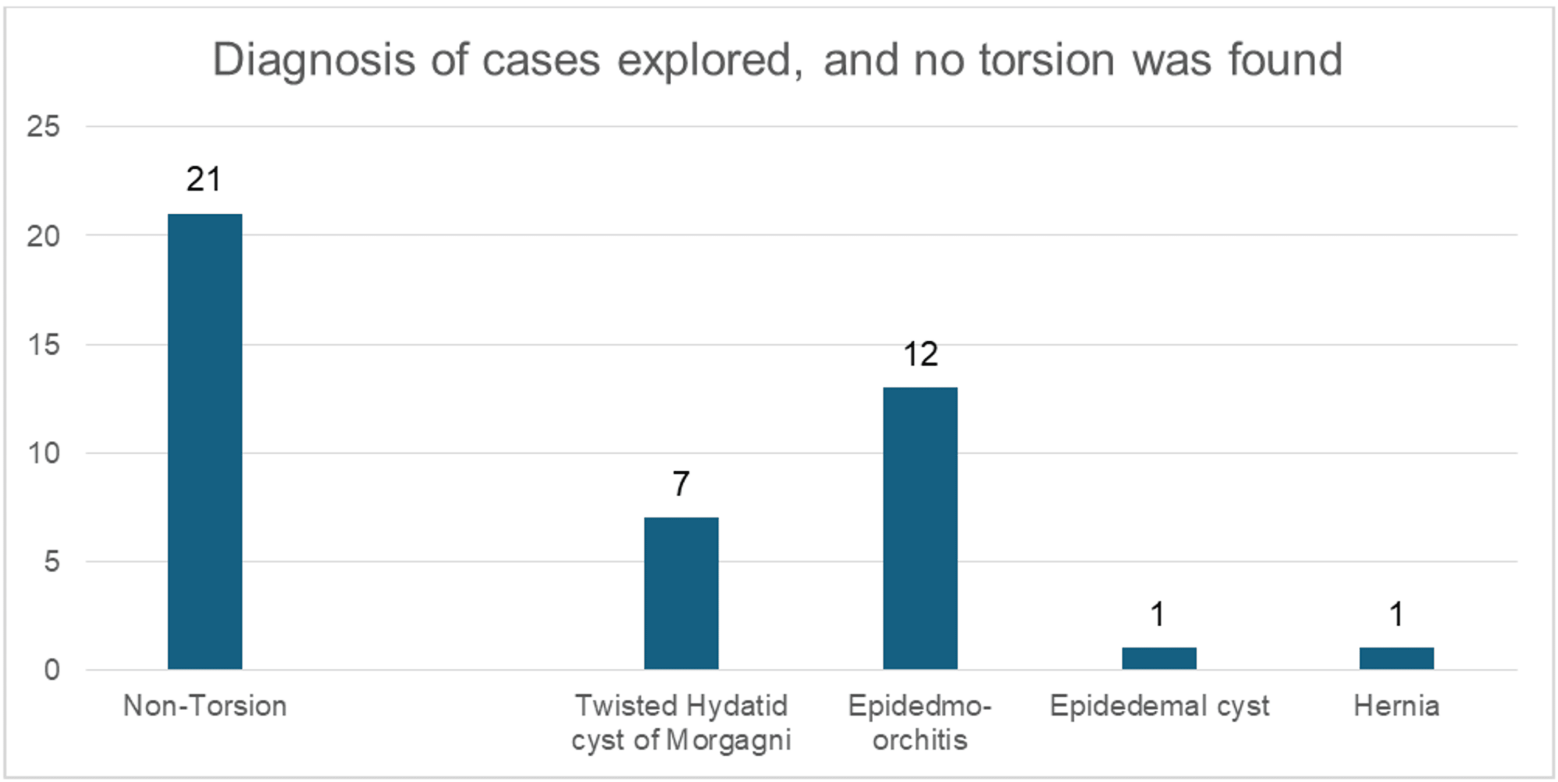
Diagnosis of cases explored, and no torsion was found

Only 38% (n=13) had confirmed TT, while 62% (n=21) had alternative diagnoses (twisted hydatid: n=7, 33%; epididymo-orchitis: n=12, 57%; epididymal cyst: n=1, 4.7%; hernia: n=1, 4.7% (Figures 2, 3)).

**Figure 4:**
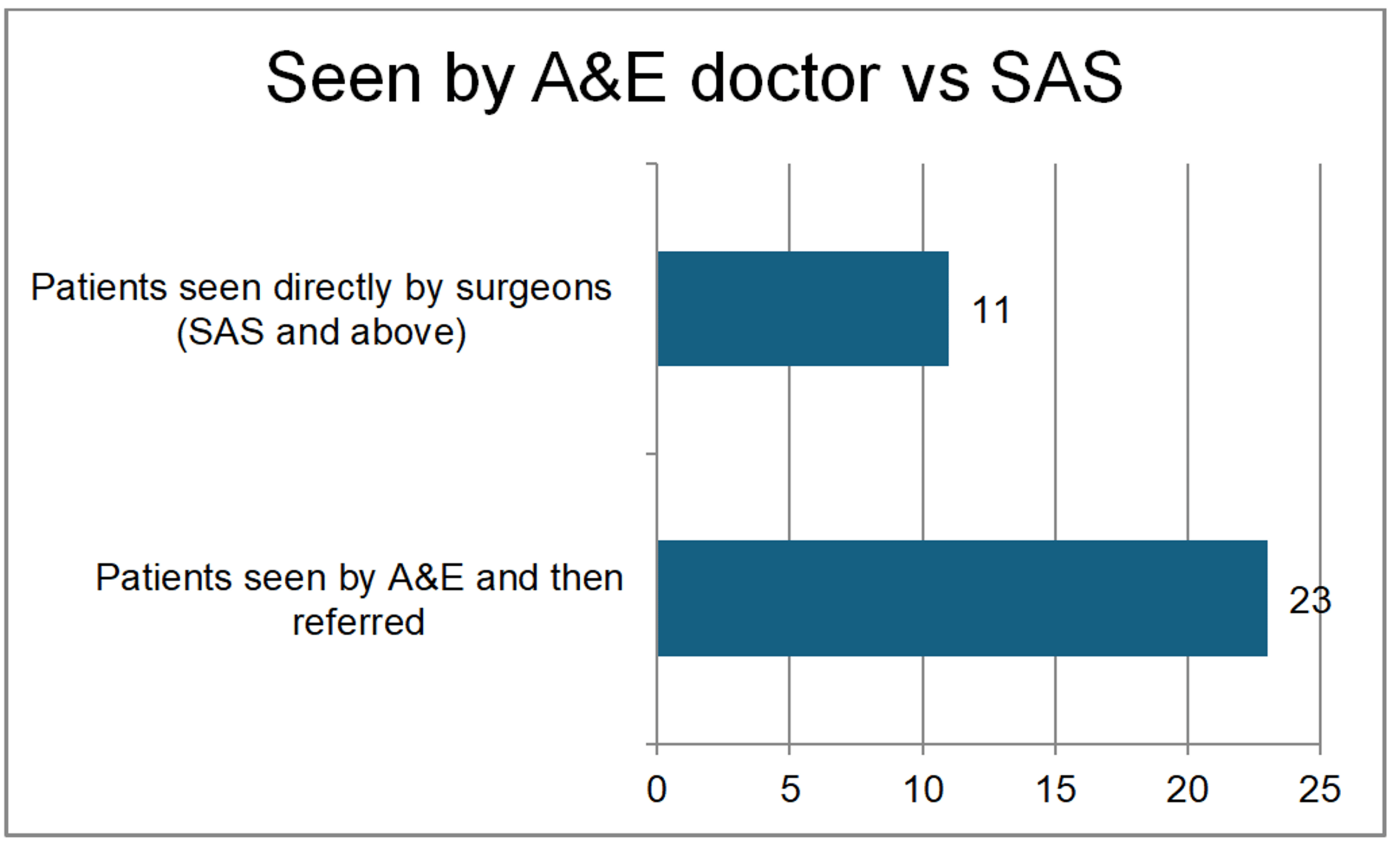
Patients reviewed by A&E doctors and referred vs. direct review by the surgical team A&E: accident and emergency; SAS: staff grade associate specialist

Only 9% (n=3) underwent surgery within the ideal one-hour window post decision, 35% (n=12) lacked documentation of critical time points (e.g., decision-to-operation), patients were seen by accident and emergency (A&E) doctors first (n=23) 67.6%, and directly referred to the surgical on-call team (n=11) 32% (Figure 4). 

**Figure 5:**
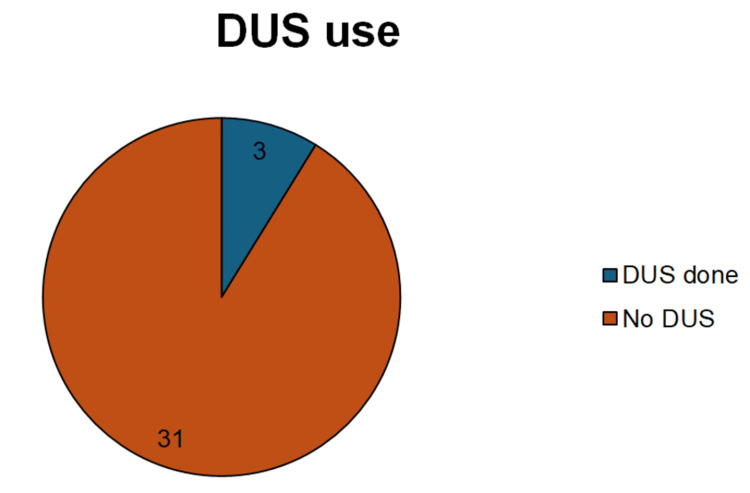
Number of patients who had DUS vs. no DUS done DUS: Doppler scrotal ultrasound

Underutilisation of diagnostic tools, as the TWIST score was documented in only 6% (n=3) cases. DUS was performed in only 9% (n=3) of cases (Figure 5).

**Figure 6:**
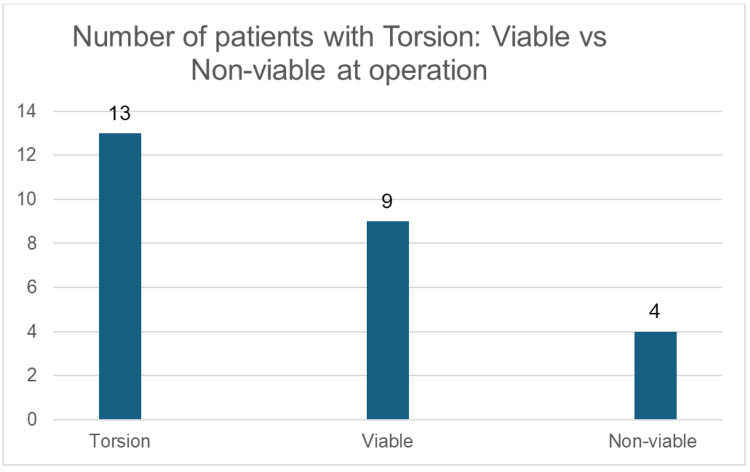
Number of patients with torsion: viable vs non-viable at operation

These findings highlight inefficiencies in risk stratification and delays in definitive management, showing the importance of a standardised protocol. Among patients who had confirmed torsion (n=13), the testis was viable in (n=9) 69% and non-viable in (n=4) 31% of the patients (Figure 6).

## Discussion

Barbosa et al. introduced the TWIST score for acute scrotal pain in 2013 [6]. Despite its efficacy, our study revealed low usage (6%) of the TWIST score at BGH, suggesting missed opportunities to reduce unnecessary explorations. The studies validated the TWIST score, showing a negative predictive value (NPV) of 98.5% for low-risk scores, effectively excluding TT. High-risk scores (≥5) correlate strongly with torsion, justifying urgent surgery [9, 10]. 

At BGH, only 9% (n=3) of patients underwent DUS, showing either underuse or systemic barriers (e.g., limited availability). Liang et al. concluded that DUS has a sensitivity of 100% and a specificity of 97.9% for detecting torsion [11]. Current evidence supports avoiding ultrasound in high-risk (TWIST ≥5) cases to prevent treatment delays [12]. Reserving ultrasound for intermediate-risk (TWIST 3-4) or equivocal presentations [13]. Gopal et al. reported that the use of DUS in low-risk and intermediate-risk patients was accurate in 95% of patients presenting with acute scrotum to exclude TT, and the use of the TWIST score with DUS led to a plummet in the annual cost to £93,000 less in one hospital [14]. 

The study findings are consistent with the NCEPOD’s guidelines (2024) [8], which emphasise surgery within four hours of symptom onset to salvage viability. European Association of Urology (EAU) guidelines (2023) [15] recommend selective ultrasound use, particularly if the presentation to hospital is more than six hours after the onset of symptoms, and the TORSAFUF (TOrsion of the Right or SAmenatic Fun / Ultrasound Factors) study (n=2922) supports ultrasound safety, but clinical suspicion is more important than DUS [16]. 

As per GIRFT children and young people, the TT pathway reported that patients with suspicion of TT should be assessed by the surgical on-call team within one hour of arrival at A&E and should be operated on within 60 minutes of the decision to operate. A TWIST score of 5 or more warrants scrotal exploration if the onset of pain is less than 48 hours, and if the TWIST score is below 5, it does not rule out TT. Ultrasound is advised if the scrotal pain’s duration is more than 48 hours, or if the pain is less than 48 hours, it’s only used to support the TT diagnosis without delaying surgical intervention, but in neonates, an ultrasound is used to exclude other differential diagnoses. After orchidopexy or orchidectomy, the patients will need urological follow-up to assess testicular atrophy and to discuss prosthesis implantation later. Effective education tools and resources for patients, parents, and schools will raise awareness of TT and the importance of early reporting to health facilities, which would improve management outcomes [7].

At BGH, delays in surgical intervention and low TWIST/ultrasound use suggest systemic gaps. Implementing a structured pathway could reduce unnecessary surgeries (currently 62% non-torsion explorations). Prioritise urgent interventions for high-risk cases. And improve documentation and compliance with guidelines. 

The proposed pathway for management of patients with scrotal pain and suspected of TT at BGH to improve care is as follows: 1. Initial assessment: Rapid TWIST scoring by emergency clinicians; high-risk (≥5 points): Immediate surgical referral without imaging; intermediate-risk (3-4 points): Expedited ultrasound (if available); proceed to surgery if positive/equivocal; and low-risk (≤2 points): Consider alternative diagnoses (e.g., infection); ultrasound if symptoms persist. 2. Operational improvements: mandate TWIST scoring for all acute scrotum presentations, fast-track surgical referrals for high-risk cases, strict time tracking starting from arrival to accident and emergency, surgeon review, prompt operative intervention, and staff training on TT recognition and documentation. 

The study has several limitations, as its retrospective design relies on medical records, which may introduce documentation biases or incomplete data. The small sample size (n = 34) from a single rural centre study makes it susceptible to selection bias. Additionally, potential confounding factors, such as variations in clinician experience or resource availability during out-of-hours periods, were not fully controlled.

## Conclusions

Integrating the TWIST score into initial assessments at BGH can improve the accuracy of TT diagnosis and reduce delays. Ultrasound can be selectively used as an adjunct in intermediate-risk cases, ideally to avoid unnecessary explorations, but in our rural setting, getting a sonographer isn’t always feasible for out-of-hours ultrasound; it’s better to have the surgical team assess urgently, deciding on exploration without delay. In order to avoid prolonging time-to-surgery, it is important to emphasise the importance of developing a standard protocol, staff education, and improved documentation for improving outcomes in this time-sensitive condition. 
